# *trans*-Double Bond-Containing Liposomes as Potential Carriers for Drug Delivery

**DOI:** 10.3390/molecules22122082

**Published:** 2017-11-28

**Authors:** Giorgia Giacometti, Marina Marini, Kyriakos Papadopoulos, Carla Ferreri, Chryssostomos Chatgilialoglu

**Affiliations:** 1 Institute of Nanoscience and Nanotechnology, N.C.S.R. “Demokritos”, 15310 Agia Paraskevi, Athens, Greece; g.giacometti@inn.demokritos.gr (G.G.); k.papadopoulos@inn.demokritos.gr (K.P.); 2 Department of Experimental, Diagnostic and Specialty Medicine, School of Medicine, University of Bologna, Via Belmeloro 8, 40126 Bologna, Italy; marina.marini@unibo.it; 3 ISOF, Consiglio Nazionale Delle Ricerche, Via P. Gobetti 101, 40129 Bologna, Italy; carla.ferreri@isof.cnr.it

**Keywords:** *trans*-phospholipids, liposomes, drug-delivery system, *trans* fatty acids, *cis*-*trans* isomerization

## Abstract

The use of liposomes has been crucial for investigations in biomimetic chemical biology as a membrane model and in medicinal chemistry for drug delivery. Liposomes are made of phospholipids whose biophysical characteristics strongly depend on the type of fatty acid moiety, where natural unsaturated lipids always have the double bond geometry in the *cis* configuration. The influence of lipid double bond configuration had not been considered so far with respect to the competence of liposomes in delivery. We were interested in evaluating possible changes in the molecular properties induced by the conversion of the double bond from *cis* to *trans* geometry. Here we report on the effects of the addition of *trans*-phospholipids supplied in different amounts to other liposome constituents (cholesterol, neutral phospholipids and cationic surfactants), on the size, ζ-potential and stability of liposomal formulations and on their ability to encapsulate two dyes such as rhodamine B and fluorescein. From a biotechnological point of view, *trans*-containing liposomes proved to have different characteristics from those containing the *cis* analogues, and to influence the incorporation and release of the dyes. These results open new perspectives in the use of the unnatural lipid geometry, for the purpose of changing liposome behavior and/or of obtaining molecular interferences, also in view of synergic effects of cell toxicity, especially in antitumoral strategies.

## 1. Introduction

**Figure 1:**
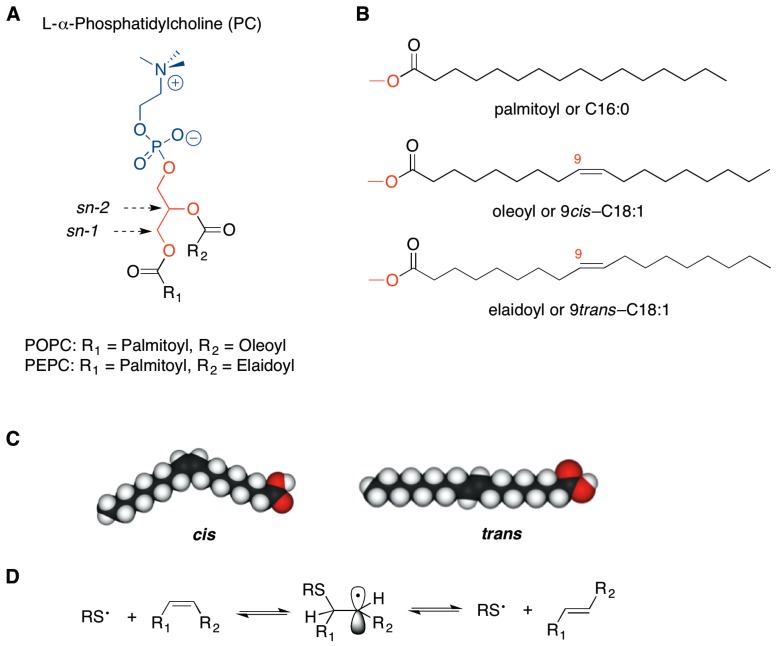
Molecular structures of l-α-phosphatidylcholine (**A**) and of fatty acid fragments (**B**); The comparison of oleic acid and elaidic acid structures to evidence the loss of the bent *cis* geometry (**C**); Reaction mechanism for the *cis*-*trans* isomerization catalyzed by thiyl radicals (**D**).

The use of vesicles called liposomes, created by the aggregation of saturated and unsaturated phospholipids (cf. Figure 1A), represented an advancement for chemical biology studies of these membrane biomimetic systems [1] and for expanding drug delivery strategies [2]. Over the years, liposomes have gained attention for carrying therapeutics, thanks to their high versatility combined with their high biological compatibility. Indeed, due to their amphiphilic properties, drugs with different partition coefficients can be incorporated into liposomes allowing control of the degradation rate and the harmful side effects. Moreover, the similarity of liposomes to biological membranes makes them non-immunogenic, physiologically inert and highly tolerated by the organism [3]. 

The variation of liposome surface and composition can help biodistribution and pharmacokinetics, promoting controlled and sustained drug release together with drug accumulation in the targeted site of action [4,5]. It is interesting to note that several strategies are combined in the so-called stimuli responsive liposomes, going from internal (biologically occurring) stimuli such as pH, temperature, redox microenvironment, to external stimuli, such as magnetic field, ultrasound, light and heat.

As far as the fatty acid residues of the membrane phospholipids are concerned (Figure 1B,C), changes of the unsaturation index and of the double bond geometry of natural fatty acids affect physical properties of the membrane bilayer, such as fluidity and permeability, with consequences on the surface interactions, protein functioning and lipid signaling [6,7,8]. In particular, we were interested in the geometry of the double bonds, given the evidence that in bacteria, the conversion from *cis* to *trans* geometry is enzymatically induced to create a membrane barrier, as a protective mechanism against increases in temperature or in toxic substance concentration of the surrounding environment [8,9,10]. In humans, such an enzymatic transformation does not occur. 

In the last two decades the occurrence of *trans* lipids has been studied in two main contexts: (a) dietary supplementation, as exogenous source of *trans* lipids in foods that have undergone industrial processes like partial hydrogenation [11] and deodorization [12]; and (b) endogenous formation by cellular stress conditions which give rise to sulfur centered radicals, highly specific and reactive for the *cis*-*trans* double bond isomerization of biological lipids (Figure 1D) [6,8,13,14]. The isomerization process has been also connected with free radical formation generated in cells by thiol-metal complexes, such as those involved in antitumoral drug mechanisms. Indeed, *trans* fatty acids (TFA) have been recently reported in membrane phospholipids of cell models treated with bleomycin, thus suggesting a participation of the lipid transformations in the toxic effects of antitumoral drugs [15]. 

The idea that TFA incorporation in the fluid mosaic of cell membranes affects their assembly properties and in vivo functions is sustained by the effect of high dietary consumption of these unnatural lipids. In fact, cell membrane incorporation of TFA is associated with the rise in several endothelial dysfunction markers—including ICAM-1 intercellular cell adhesion molecules, VCAM-1 vascular cell adhesion molecules and E-selectin—and with the loss of endothelium-mediated vasodilatatory response [16]. 

So far, the *trans* geometry of the cell membrane fatty acid pool has been poorly addressed. Some preliminary differences between *cis*- and *trans*-containing mono-unsaturated liposomes, regarding diameter and fluidity properties, were found, which could be related to the molecular shapes [17,18]. Whether such variations induced by *trans*-phospholipid might be used for imparting differences in drug encapsulation efficiency or release profile, influencing liposome stability and drug leakage, is not known so far. 

In this study we report the effects induced by the presence of *trans*-phospholipids on the size, zeta (ζ) potential and stability of liposomal formulations. The encapsulation efficiency and the in vitro release of two different dyes, rhodamine B and fluorescein (commonly used to study the properties of different nanocarriers [19,20]), were compared in liposome formulations containing known components (such as cholesterol, phosphatidyl-choline and -ethanolamine, cationic surfactant) and differing for their *cis*- or *trans*-phospholipid geometry. In perspective, the results suggest that *trans*-phospholipids may be interesting players in antitumoral formulations, because of their ability to influence the liposome behavior; the synergy of the drug effect with the *trans* geometry of phospholipids can be envisioned as an innovative antitumoral strategy. It is worth mentioning that the formation of *cis* double bond (stearic to oleic acid transformation) is a crucial enzymatic pathway involved in tumorigenesis [21,22].

## 2. Results and Discussion

*trans*-Phospholipids were prepared by a synthetic procedure using gamma irradiation (Cobalt-60 source) on the natural 1-palmitoyl-2-oleoylphosphatidylcholine (POPC), having the natural oleic acid (9*cis*-C18:1) as mono-unsaturated chain, in the presence of 2-mercaptoethanol [13,14]. The thiyl radical, generated from the thiol, catalyzed the conversion of oleic moiety to the corresponding geometrical isomer (9*trans*-C18:1), see Figure 1D. More specifically, the product of the reaction was a *trans* fatty acid-containing phosphatidylcholine mixture, composed by 60% by 1-palmitoyl-2-elaidoylphosphatidylcholine (PEPC) and 40% POPC. The *trans* content was established after purification and conversion of a small sample fraction to the corresponding fatty acid methyl ester (FAME) followed by gas chromatography (GC) analysis. This phospholipid mixture was named 60-PEPC. Another phospholipid mixture containing an intermediate percentage of elaidic acid, i.e., consisting of 30% PEPC and 70% POPC, was prepared and named 30-PEPC. 

Multilamellar vesicles (MLVs) were obtained from 0.01M phosphate buffered saline (PBS) suspension of 10 mM *cis*- and *trans*-containing phospholipids. The MLV suspensions were then downsized by extrusion technique using 100 nm polycarbonate membrane filters to form large unilamellar vesicles (LUVETs), that are more suitable for drug delivery purposes. Liposomes were analyzed by Dynamic Light Scattering (DLS) that gives information about the size distribution, the homogeneity and the surface charge of the liposomal suspensions, as well as about aggregation or disruptive phenomena.

### 2.1. Effect of the Formulation on Size and ζ-Potential

**Figure 2:**
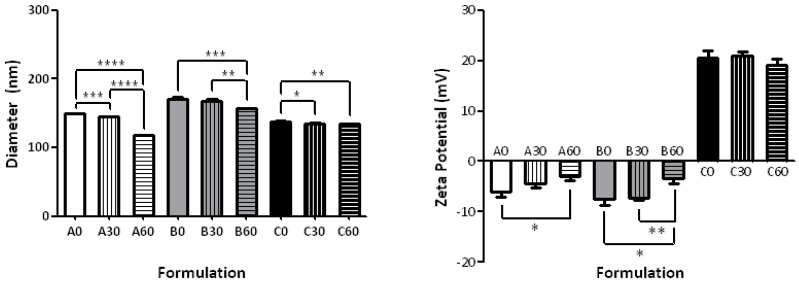
Size distribution and ζ-potential of various liposomal formulations (taken from Table 1). Significant differences (*p* value) are reported as follow: (*) *p* ≤ 0.05, (**) *p* ≤ 0.01, (***) *p* ≤ 0.001, (****) *p* ≤ 0.0001.

The results of the particle sizing and ζ-potential measurements of the various liposomal formulations are summarized in Table 1 and graphically represented in Figure 2. The simplest formulation to investigate the differences due to the percentages of *cis* and *trans* bonds consisted of the natural POPC in comparison with formulations having 30-PEPC and 60-PEPC. A decrease in size was observed as the *trans* phospholipid percentage increased (Table 1—Formulation A0, A30, A60). A significant drop in size, from 149.1 ± 0.18 nm to 117.4 ± 0.55 nm (*p* ≤ 0.0001), was observed by replacing POPC with 60-PEPC. As for the Polydispersity Index (PDI), that provides information about the size distribution of the suspensions, and for the average surface charge of the particles, indicated as ζ-potential, no large differences were noticed among the three formulations. 

The inclusion of cholesterol in the formulation, used in a molar ratio of 70:30 phospholipid/cholesterol (7 mM phospholipids and 3 mM cholesterol) [23], induced an increase in size compared to natural phosphatidylcholine liposomes (Table 1—Formulations B0, B30, B60). This is one of the expected changes due to the cholesterol interactions within the lipid bilayer, which are known to influence lipid order, bilayer width and properties, such as permeability and fluidity [24,25,26]. The presence of 60-PEPC (B60), but not 30-PEPC (B30), led to a decrease in size and PDI and an increase in ζ-potential compared to POPC (B0). The decrease caused by the *trans* geometry to the size and PDI in the presence of cholesterol is worth of note, since at the 60% PEPC concentration in the bilayer the cholesterol effect seems to be annihilated. This aspect could suggest an interesting connection with the role of cholesterol in the *cis*-*trans* isomerization process in bacteria, as protection against environmental changes [9,10]. In all cases the presence of cholesterol decreased the ζ-potential value that is a known effect such as in case of Na^+^ ion binding with the lipid head group [27], although in the presence of 60-PEPC the decrease is much less. 

Due to the expanding field of gene therapy, that requires positively charged liposomes to carry negatively charged oligonucleotides, a cationic formulation containing the cationic surfactant, myristoyl trimethylammonium bromide (MTMAB) was also tested. In particular, formulations containing a mixture of POPC/DPPC/CHOL/POPE/MTMAB 25:25:20:15:15 or the analogous 30-PEPC or 60-PEPC in replacement of POPC, were prepared (Table 1—Formulation C0, C30, C60). Components able to synergize in the enhancement of the membrane stability were chosen in such formulations. In fact, (a) 1,2-dipalmitoylphosphatidylcholine (DPPC) is important to preserve the thermostability of liposomes in physiological conditions, due to its high transition temperature [28,29]; (b) 1-palmitoyl-2-oleoylphosphatidylethanolamine (POPE) is a phosphatidylethanolamine (PE) that can form stable bilayers at physiological pH when intercalated with other amphiphilic molecules, although, in an acidic environment, protonation of its carboxylic group causes the shift to the inverted hexagonal phase that destabilize the liposome structure. For this reason, PE is widely used in cancer gene therapy since it can lead to lysosome destabilization and selective drug release in the acidic extracellular tumour environment [30,31]; (c) MTMAB is a quaternary surfactant able to enhance the cellular internalization and the DNA transfection in gene therapy [32,33]. Indeed, cationic liposomes appear to be the safest and cheapest alternative to viral vectors for protecting DNA from enzymatic degradation and efficiently delivering high molecular weight molecules without provoking dangerous immunological responses [34,35,36]. The diameter range of the three cationic formulations was smaller compared to the above-mentioned ones and their ζ-potentials were close to +20 mV (Figure 2). The presence of surface positive charges, as seen in this case, induces electrostatic repulsions that prevent formation of aggregates or flocculation of liposomes. Moreover, positive net charges could promote the interaction of liposomes with cells, due to the asymmetric membrane lipid distribution of phosphatidylserine that confers a negative charge to cell membranes [37]. Very small values of PDI (<0.15) were recorded for POPC and 60-PEPC formulations, which indicates narrow monodisperse systems. 

In general, it was observed that, as the *trans* content in the membrane increases, the mean particle size decreased. Only small differences with the *cis* formulations were observed when 30-PEPC was used (cf. Figure 2). Particle diameters greater than 100 nm, the size of the filter pores, can be explained by the interaction of the phospholipid heads with buffer ions in the surrounding environment; on the other hand, the fact that the liposome size is still <200 nm is important for the escape from complement recognition and uptake by the mononuclear phagocytic system (MPS) [38]. Smaller sizes are desired to exploit the passive targeting due to the so-called enhanced permeability and retention effect (EPR) where extravasation of liposomes into solid tumours is essential [39]. The accumulation of liposomes in tumours is size-dependent, as tumour capillaries have larger pores (100 to 700 nm in diameter) than normal blood vessels (typically <50 nm). Thus, liposomes between 90 and 200 nm in diameter are able to selectively penetrate tumour capillaries [40,41].

### 2.2. Effect of the Formulation on Stability (Size vs. Time)

Since formulations with 60-PEPC gave better results in terms of size and PDI compared to the 30-PEPC formulations, further studies were performed to understand the effect of *trans*-inclusion in liposomal membrane in comparison with *cis*-analogues. Stability studies were conducted by storing the formulations at temperatures of 22, 37 and 45 °C for 72 h and by analyzing them by DLS, GC and thin layer chromatography (TLC). 

**Figure 3:**
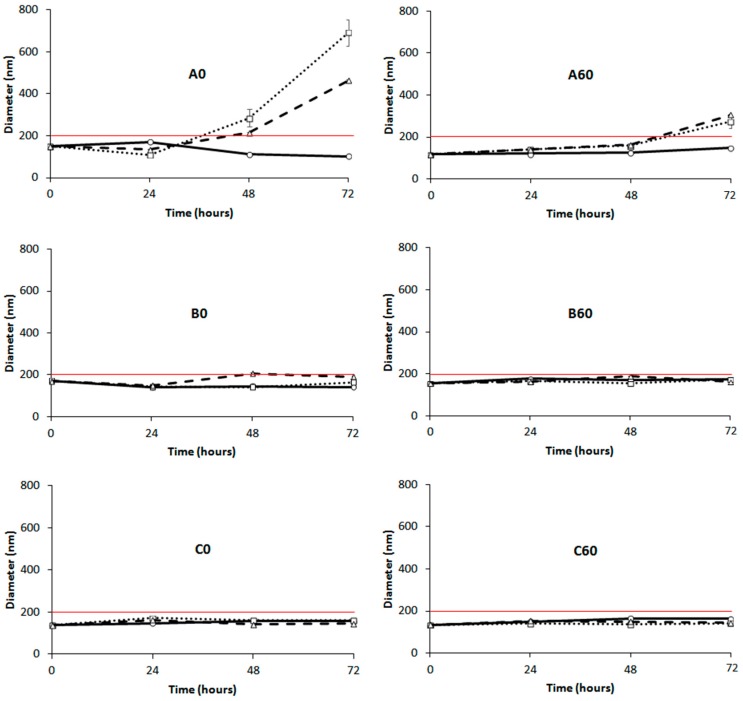
DLS measurements of liposomes at 22 °C (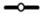), 37 °C (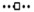) and 45 °C (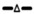) versus time. (**Left panels**): POPC formulation A0, B0 and C0; (**Right panels**): 60-PEPC formulation A60, B60 and C60. The red line shows the size threshold of 200 nm.

Figure 3 shows the DLS results for the formulations A0, B0 and C0 using commercially available POPC (left panels) and for the analogous formulations using 60-PEPC (right panels). The formulation A0 showed aggregation phenomena when stored at 37 and 45 °C. The formulation A60 reduced this tendency but the aggregation, driven by the neutral charge of the phospholipid employed for this preparation, could not be avoided [42]. Addition of cholesterol improved the stability of the formulations at all the temperatures, both in the presence of POPC (B0) and 60-PEPC (B60). Good stability was also observed when the cationic formulation was tested, regardless of the inclusion of *trans* lipids (C0 and C60). Reasonably, the presence of surface charges reduced the tendency of liposomes to flocculate and form bigger particle aggregates. 

TLC and GC analyses, useful to detect formation of by-products or oxidative consumption of unsaturated lipids, did not evidence any alteration of lipid composition and concentration (data not shown).

### 2.3. Atomic Force Microscopy

**Figure 4:**
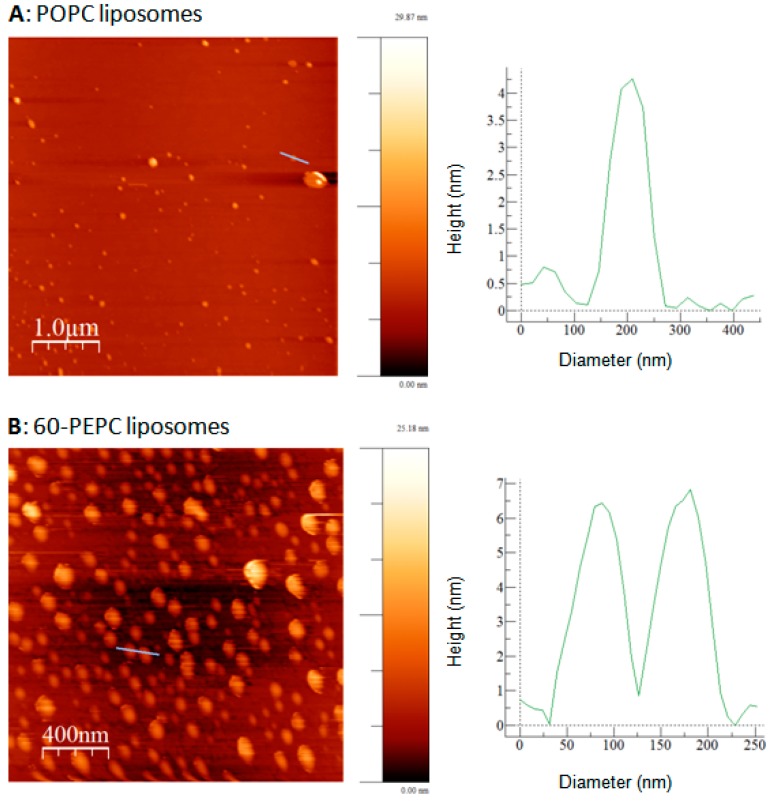
Representative AFM images (on the left) and cross-section probles (on the right) of POPC (**A**) and 60-PEPC (**B**) liposome acquired with tapping mode as described in Experimental Section.

POPC and 60-PEPC liposomes were analyzed by atomic force microscopy (AFM), also known as scanning force microscopy (SFM), which is one of the most common techniques for liposome description [43,44]. AFM provides high nanoscale spatial resolution combined with minimal sample preparation that preserves unaltered the sample characteristics. Indeed, compared to scanning electron microscopy (SEM) and transmission electron microscopy (TEM), which require complex sample preparation such as chemical fixing or metal coating, AFM leaves unaltered the surface to be scanned [45,46]. High sensitivity is reached using the tapping mode that, through small oscillations of the cantilever, permits to collect information about nanocarrier morphology and thickness. As a result, differences in rigidity can be observed and numerically expressed by the height/diameter ratio [47]. Measurements of approximately 10 liposome diameters were performed for each sample. The results are provided (as mean of triplicate measurements) in Figure 4 and in Table 2 a representative AFM image is shown for POPC and 60-PEPC liposomes. 

Both formulations showed spherical shape with homogeneous distribution. POPC liposomes resulted to be larger in comparison with 60-PEPC. Considering the liposome height, POPC liposomes resulted to be collapsed on the support. This property can be expressed by the height/diameter ratio that gives general information about the liposome rigidity. Indeed the higher the height/diameter ratio, the more rigid the system is.

These scanning probe microscopy measurements confirmed the different properties of *trans*-containing liposomes compared to *cis*-containing ones, as previously shown by other techniques, detailing the features of dimensions and rigidity.

### 2.4. Effect of the Procedure on the Encapsulation Efficiency

Four different conditions were tested to ensure the maximal drug encapsulation yield, as summarized in Table 3. The conditions were optimized on the POPC formulations (A0, A30, and A60) using rhodamine B as drug model. Since rhodamine B is an aminoxanthene with amphiphilic characteristics, it was dissolved in the aqueous solution and used to rehydrate the phospholipid film. Briefly, the effect of vortexing at 22 °C and 40 °C, followed by sonication or freeze-annealing-thaw cycles was studied. The molar ratio between the dye and the lipids (D/L) played a crucial role in the successful encapsulation of the fluorescent molecule. Four different D/L of 1:1, 1:10, 1:50, and 1:100 were tested.

**Figure 5:**
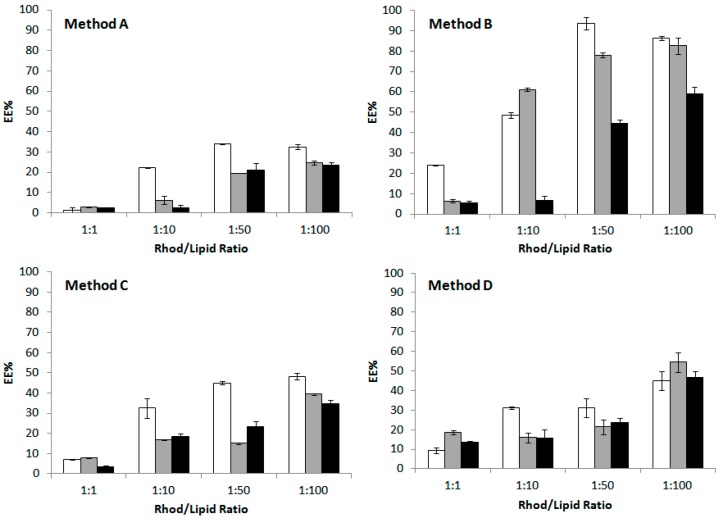
Effect of the encapsulation technique on the encapsulation efficiency (EE) of rhodamine B in liposomes formulations A0 (white), B0 (grey) and C0 (black). Encapsulation of rhodamine B was carried out by vortexing the suspension for 10 min at 22 °C (**Method A**), or at 40 °C followed by 2 × 5 min sonication cycles (**Method B**). The effect of two (**Method C**) or five (**Method D**) cycles of freeze-annealing-thaw technique after (**Method A**) on EE was also tested. Error bars show the differences found in the experiments run in triplicates.

Results are reported in Figure 5. With the lowest D/L ratio, when the suspensions were vortexed at room temperature for 10 min at maximum speed (Method A), only 20% to 40% of dye encapsulation efficiency (EE) was observed.

Warming up of the buffer to 40 °C prior to the hydration step, followed by two brief sonication cycles of 5 min (Method B), resulted in a significant improvement of rhodamine B loading in the liposomal vesicles. Among the three formulations, POPC liposomes (A0) were the most efficient in including the dye in the inner aqueous volume. Probably, the presence of cholesterol decreased the encapsulation efficiency of the fluorescent molecule since it induces the membrane condensation and directly competes with rhodamine B in the bilayer assembly [48]. 

Another important technique often used to enhance drug encapsulation into nanocontainers is the freeze-thaw procedure [49,50], but high variability in the number of cycles has been reported in literature [51,52]. In this work, the sample underwent two (Method C) or five (Method D) freeze-anneal-thaw cycles (freezing to −196 °C with liquid nitrogen, annealing at 0 °C for 30 min in ice bath, thawing at 40 °C in a water bath), since drug diffusion occurring in the frozen state and fusion/destabilization of liposomes led to higher EE compared to the traditional method [53]. Apparently, an increase in the number of freeze-anneal-thaw cycles gave higher percentages of encapsulated dye but not as much as sonication. Probably, the steric hindrance of the molecule to be encapsulated does not favour its entrapment and interaction with the lipid fraction. 

It is worth to underline that the molar ratio between dye and lipid was very important, since by varying the D/L it is possible to optimize the drug loading and release from liposomal formulation [54]. In this set of experiments, 1/100 was chosen as the best D/L not only because it allowed to reach 60 to 90% of EE using Method B, but also because using higher D/L ratio could induce drug precipitation and membrane bilayer destabilization [55]. In these formulations the total lipid concentration is 10 mM (see Experimental Section).

**Figure 6:**
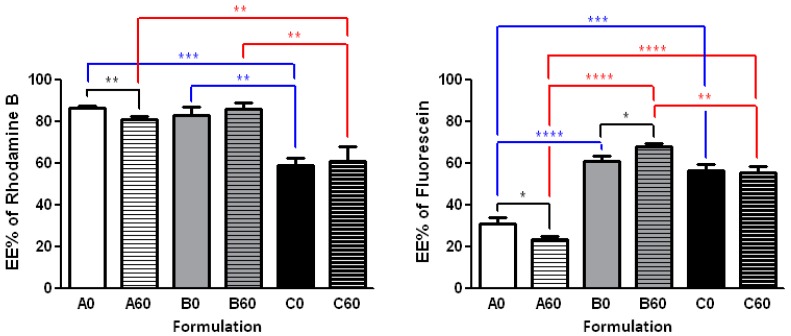
Encapsulation efficiency (EE) of rhodamine B (**Left panel**) and fluorescein (**Right panel**) in liposomes formulations A0, B0 and C0, and the corresponding formulations containing 60-PEPC (A60, B60 and C60 respectively). The encapsulation procedure was carried out at 40 °C using two sonication cycles of 5 min each (Method B) and 1:100 dye/lipid ratio. Experiments were run in triplicate. Results are shown as mean EE% ± sd. Significant differences (*p* value) are reported as follow: (*) *p* ≤ 0.05, (**) *p* ≤ 0.01, (***) *p* ≤ 0.001, (****) *p* ≤ 0.0001. Differences between *cis* and *trans* containing liposomes having the same formulation (black), but also between *cis* containing formulations (blue) and *trans* containing formulations (red) are reported.

In the light of these results, Method B, that combines temperature and sonication effects, and a 1:100 dye/lipid ratio, for the best encapsulation efficiency, was chosen as the most favorable encapsulation conditions to be applied with the 60-PEPC containing formulations, i.e., A60, B60 and C60 (Figure 6). Comparing the effects of the three different formulations in the EE of rhodamine B, it is clear that only the charged liposomes were less prone to incorporate the dye (Figure 6, left panel). Comparing the effects of the *trans* geometry of the phospholipid (60-PEPC), the reduction of the EE was significant in case of the simplest formulation (cf., A0 vs. C0, A60 and B60 vs. C60), indicating the direct effect of the packing of these liposomes compared to simple phospholipids and cholesterol assemblies. 

The same procedure was used to encapsulate a different dye, fluorescein (Figure 6, right panel), an oxyxanthene insoluble in water. Here, the effect due to the hydrophobic nature of this dye is clear, leading in all formulations to a diminished incorporation compared to rhodamine B. However, both the addition of cholesterol and of the charged lipids significantly increased EE (cf., A0 vs. B0 and C0). The effects of the *trans* geometry (60-PEPC) on these liposomes is again evident in the simplest composition (A0 vs. A60), whereas cholesterol brought a significant increase of EE for the *trans* lipid, confirming the spacing effect created by the sterol presence in the bilayer (B0 vs. B60). The charged surface of liposomes in the case of C0 and C60 resulted into an intermediate effect on EE. The presence of cationic lipids in the “C” formulation can also have a stabilizing effect on the incorporation of fluorescein, which is negatively charged at pH 7.4 (pK_a1_ = 4.45; pK_a2_ = 6.8).

### 2.5. Effect of the Liposomal Formulation on the Release

The in vitro release of rhodamine from the liposomal formulations at 22 °C and 37 °C was tested. Dialysis bags containing 0.5 mL of dye-containing liposomes were dialyzed against 10 mL of PBS 1 X under continuous stirring. A solution of pure rhodamine B dissolved in PBS, was used as a control to show the ability of liposome to give a controlled and sustained release over time. 

**Figure 7:**
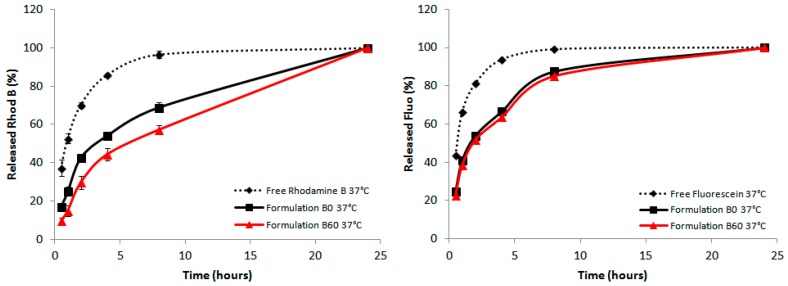
In vitro release of rhodamine B (**Left panel**) and fluorescein performed at 37 °C (**Right panel**). The POPC containing formulations (in black) were compared with the corresponding 60-PEPC containing ones (in red). The release profile of free rhodamine B and free fluorescein (dashed lines) is also reported.

In all cases the dialysis of the free dye was evidently different from the encapsulated dye formulations. All preparations reached the same dye release in the end of the process, and no differences were seen at 20 °C (data not shown). Instead, at 37 °C the B formulation gave the best effect of retardation when 60-PEPC is present as shown in Figure 7. In the formulations A and B the releases detected at the first hours were always reduced in the 60-PEPC liposomes, whereas in cationic liposomes these effects are lost. This could be due to the different partition coefficient of the dyes used for the experiments. It is possible that fluorescein, having hydrophobic characteristics, is localized in the proximity or in the depth of the double lipid layer and this causes it to be less retained by the liposomal structure. On the contrary, rhodamine B, which has amphiphilic characteristics, can be localized both in the internal cavity created by the liposomal bilayer and the depth of the liposome membrane itself and for this reason it is released slower than fluorescein.

The data presented here using dyes can be extrapolated to drug encapsulation and release behaviors, highlighting the role of *trans*-containing liposomes as potential carriers with additional properties compared to the known phospholipid formulations. Resistance and release experiments, either using human plasma and cell cultures, will be required and our results encourage further work to take advantage of the lipid diversity, also considering the double bond geometrical features. 

## 3. Experimental Section

### 3.1. Materials

1,2-Dipalmitoylphosphatidylcholine (DPPC), 1-palmitoyl-2-oleoylphosphatidylcholine (POPC), and 1-palmitoyl-2-oleoylphosphatidylethanolamine (POPE) were purchased from NOF American Corporation (Irvine, CA, USA). Sodium sulfate anhydrous, phosphate-buffered saline (PBS) tablets, cholesterol and myristoyl trimethylammonium bromide (MTMAB) were purchased from Sigma Aldrich (St. Louis, MO, USA). Rhodamine B was purchased from Janssen Chimica (Beerse, Belgium) and fluorescein was purchased from Merck (Darmstadt, Germany). 2-Mercaptoethanol (98% purity) was purchased by Alfa Aesar (Karlsruhe, Germany). 30% NH_4_OH aqueous solution was purchased from Panreac Quimica SA (Barcelona, Spain). Potassium hydroxide was purchased from Riedel De Haen AG (Seelze, Hannover, Germany). 2-Propanol (Analytical grade) and chloroform (HPLC grade) were purchased from Fisher Scientific (Atlanta, GA, USA). Methanol and *n*-hexane, were purchased from Macron Fine Chemicals (Center Valley, PA, USA). Silica gel thin-layer chromatography was performed on Merck silica gel 60 plates (0.25 mm thickness) and the spots were detected by spraying the plate with cerium ammonium sulphate reagent. Standard methyl ester references (FAME mix C14-C22 and 37 component FAME mix) were commercially available from Supelco (Bellefonte, PA, USA) and were used without further purification. Trans FAME references were synthesized according to previously reported protocols [14,17]. 

### 3.2. Methods

#### 3.2.1. Synthesis and Purification of PEPC

A solution of 1-palmitoyl-2-oleoylphosphatidylcholine, POPC (60 mg, ca. 0.079 mmol) in 2-propanol (4 mL) was placed in a vial and degassed with argon for 20 min. Then, 2-mercaptoethanol (2.73 mg; 0.035 mmol) was added, and the solution was irradiated for 1 h (dose rate = 2.84 Gy/min). The crude of the reaction was purified to pure phospholipids (59 mg; 0.077 mmol; 98% yield) using the chromatographic procedure described in literature [17]. Briefly, the solvent was evaporated, the crude of the reaction was dissolved in chloroform and charged on a silica column conditioned with chloroform/methanol 8:2. The sample was collected in one fraction procedure using chloroform/methanol/water/30% aqueous NH_4_OH 20:10:0.4:0.2 mixture as mobile phase. The purity of phosphatidylcholine was verified by TLC using the above specified eluent (Rf = 0.5). A small aliquot underwent GC analysis to determine the *trans* isomer content. In the product, 60% of oleic acid was converted in the corresponding *trans* isomer, elaidic acid. To this phosphatidylcholine mixture was given the name of 60-PEPC.

#### 3.2.2. Liposome Preparation

LUVETs were prepared using the hydration-extrusion technique [56]. Briefly, POPC (76 mg; 0.1 mmol) or a mixtures of phospholipids with the same molarity were dissolved in CHCl_3_/MeOH 2:1 until a clear lipid solution was obtained. The organic solvent was removed using a rotary evaporator to yield a homogeneous lipid film on the sides of a round bottom flask. The lipid film was thoroughly dried to remove residual organic solvent by placing the vial or flask on a vacuum pump for 1 h. The dried lipid film was left to hydrate for 30 min in PBS and then vortexed for 10 min until a milky mono-phasic solution containing multi-lamellar vesicles (MLVs) was obtained. For high transition lipids, PBS 1× was warmed up above the phase transition temperature (Tm) of the lipid with the highest Tm before adding to the dry lipid, and all the steps that follow were done under the same conditions. Once a stable suspension was formed, MLVs were downsized to LUVETs using a LiposoFast hand extrusion device (Avestin Inc., Ottawa, ON, Canada) equipped with 100 nm pore size polycarbonate filters through which the lipid suspension was passed 19 times, controlling the temperature with a heat block when required. The resulting suspension was used in the encapsulation and release experiments with the same molarity (i.e., 10 mM).

#### 3.2.3. Dye Encapsulation

Rhodamine B (MW 479.02 g/mol) and fluorescein (MW 332.31 g/mol), were chosen as inexpensive and common luminophore drug models. Starting from a 10 mM total lipid concentration, 1:1, 1:10, 1:50 and 1:100 dye:lipid ratio were tested in order to determine the optimal encapsulation conditions. Lipids were first dissolved in chloroform then dryed to form a thin lipid film using the rotary evaporator and left under vacuum for 30 min. 10 mM stock solutions of rhodamine B (in PBS) and fluorescein (in MeOH) were used to prepare the dye-containing diluted solutions used to rehydrate the thin lipid film. In the case of rhodamine B, the diluted solution could be immediately used to rehydrate the lipid film, while fluorescein solution in MeOH was added to the lipid film prior to the vacuum step in order to remove any trace of organic solvent. The lipid suspension was left 30 min to hydrate and then vortexed for 10 min. When freeze-annealing-thaw cycles were required, the samples were frozen at −196 °C using liquid nitrogen (3 min), left to anneal at 0 °C in an ice bath (30 min) and then thawed at 40 °C in a liquid bath (30 min). Four different procedures were tested. Method A: samples underwent 10 min vortexing performed at 22 °C; Method B: the vortexing step of 10 min duration was carried out at 40 °C, followed by two sonication cycles, each one of 5 min; Method C: after 10 min vortexing at 22 °C, samples underwent two freeze-annealing-thaw cycles; Method D: samples were vortexed for 10 min at 22 °C and then subjected to five freeze-annealing-thaw cycles. The non-encapsulated dye was removed by sequential centrifugations (18,000 *g* × 4 °C × 30 min) and washes with fresh PBS until a transparent supernatant was obtained. Rhodamine B concentration was determined by measuring its absorbance at 553 nm using a V-560 UV/Vis spectrophotometer (Jasco, Easton, MD, USA). The dose-response curve was linear (*r* = 0.999) in the concentration range of 0.1–10 µM. Fluorescein concentration was determined by measuring its absorbance at 491 nm and its fluorescence at 512 nm, using a FLUOstar OPTIMA spectrophotometer (BMG Labtech GmbH, Ortenberg, Germany). The dose-response curve was linear in the concentration range of 0.5–100 µM in absorbance (*r* = 0.9994) and 0.01–5 µM in fluorescence (*r* = 1). The measurements were recorded both in PBS 1× and in 30% ethanol. 

#### 3.2.4. Encapsulation Efficiency (EE%)

After removal of the non-encapsulated dye, the pellet of dye-containing liposomes was dissolved in 30% ethanolic aqueous solution and thoroughly sonicated in order to fully disrupt the liposome pellet and release the encapsulated dye (Direct method). This method was preferred over the commonly used detergent treatment with 10% Triton × 100 because this latter one induced a significant quenching in the fluorescence. The encapsulated amount of dyes was confirmed by measuring the not encapsulated dye (Indirect method) using the following equation:

(1) $$Encapsulation\;Efficiency\;\left(EE\%\right)=\frac{\left(Initial\;dye\right)-\left(Free\;dye\right)}{\left(Initial\;dye\right)}\times 100$$

#### 3.2.5. In Vitro Release 

Cellulose dialysis membranes with a MWCO of 12–14,000 Daltons and 28.6 mm diameter (Visking Medicell International, London, UK) were hydrated using PBS 1× 0.5 mL of the dye-loaded liposome suspension (10 mM lipids, pH 7.4) was placed into the dialysis bag, which was then transferred into a beaker containing 10 mL of PBS 1×. The beaker was put on a magnetic stirrer and kept continuously under stirring. The dye release was monitored at 22 and 37 °C to simulate storage and physiological conditions, respectively. For comparison, a solution of free dye dissolved in PBS was analysed under the same conditions. Samples of 1 mL volume were withdrawn at fixed time intervals (30 min, 1, 2, 4, 8 and 24 h) and analyzed by UV/Vis or fluorescence spectroscopy. 

#### 3.2.6. Lipid Extraction and GC Analysis

Liposome phospholipids were isolated using the well-established Folch Method [57]. Briefly, the liposomal suspension in PBS 1× was treated with 2:1 chloroform:methanol (3 × 2 mL) mixture. The organic phase was collected and dried using anhydrous sodium sulphate, evaporated and left under vacuum for 30 min. A small fraction was analyzed by thin layer chromatography (eluent: chloroform/methanol/water 65:25:4) to check the purity of the phospholipid fraction, according to published procedure [58]. The residue was treated with a 0.5 M solution of KOH in methanol (1 mL) and left under stirring for 10 min at room temperature to convert the fatty acid residues of the phospholipids into their corresponding fatty acid methyl esters (FAME). The reaction was quenched using water (1 mL) and FAMEs were extracted using *n*-hexane (3 × 2 mL); the organic phase was collected and dried with anhydrous sodium sulphate. The solvent was eliminated by evaporation using a rotary evaporator, and the thin white film of the FAME was subsequently dissolved in a small volume of n-hexane and injected into the GC. A 7890B gas chromatograph (Agilent, Santa Clara, CA, USA) equipped with a flame ionization detector and a DB-23 (50%-cyanopropyl)-methylpolysiloxane capillary column; 60 m, 0.25 mm i.d., 0.25 µm film thickness) was used for the analysis. The initial temperature was 165 °C, held for 3 min, followed by an increase of 1 °C/min up to 195 °C, held for 40 min, followed by a second increase of 10 °C/min up to 240 °C, held for 10 min. The carrier gas was hydrogen, held at a constant pressure of 16.482 psi. All the methyl esters were identified by comparison with the retention times of standard references either commercially available or obtained by synthesis, as described elsewhere [6].

#### 3.2.7. Dynamic Light Scattering (DLS)

Hydrodynamic diameter and ζ-potential of LUVET were measured using the DLS technique (Malvern Instruments Series NanoZS with a detection angle of 173°, Malvern Instruments, Malvern, UK). The optimal concentration for size distribution was 1 mM, while 0.1 mM concentration was used to measure ζ-potential. All measurements were recorded at 25 °C. In the present study, the data reported are the mean values obtained from three measurements, each one including 10 runs of 10 s. The polydispersity of the sample, a reverse parameter to describe the homogeneity of the system, is reported as polydispersity index (PDI).

#### 3.2.8. AFM Analyses

Morphological characteristics were monitored using Atomic Force Microscopy (AFM). Tapping mode was chosen as image recording parameter. The probes used were RTESPA-300 model (Bruker, Billerica, MA, USA) unmounted Si (n-type doped Sb) with Nominal constant 40–80 N/m and resonance 300–400 kHz. 20 µL of a 10 mM liposome solution were deposited on a mica support. After 2 min of incubation at room temperature, the excess of liquid was removed from a corner using a Kimtech tissue. Samples were analyzed in triplicates. Results are expressed as mean ± SD.

#### 3.2.9. Statistical Analysis

Results are given as means ± SD. All data reported are presented as mean or percentage ± standard deviations (SD, *n* = 3). Statistical significance (*p* values) of the results was calculated by unpaired two-tailed Student’s *t*-test using GraphPad Prism version 6.01 for Windows (GraphPad Software, San Diego, CA, USA). Differences were considered to be statistically significant if *p* < 0.05.

## 4. Conclusions

The results presented herein show the importance of the *trans* geometry of double bonds for the properties of liposomes, not only for their formation and structure, but also for the incorporation and release of molecules, using dyes as representative compounds. The presence of the unnatural lipid geometry compared to the natural *cis* lipids influences these processes as shown in our experiments, which suggests to deepen such effects in case of biological membranes where *trans* lipids can derive from endogenous or exogenous sources. Importantly, since the presence of *cis* geometry is crucial for the membrane fluidity contribution in cell signalling, the molecular interference given by *trans* lipids can be further developed for synergic effects in antitumoral strategies.

## Figures and Tables

**Table 1:** Average size diameter, polydispersity index and ζ-potential of various liposomal formulations prepared in PBS as MLV and extruded through a 100 nm polycarbonate filter. The values are given as mean ± SD. Each experiment was performed in triplicate.

Formulation	Composition	Size (nm)	PDI	ζ-Potential (mV)
A0	POPC	149.1 ± 0.18	0.18 ± 0.01	−6.03 ± 1.05
A30	30-PEPC	144.45 ± 0.78	0.11 ± 0.06	−4.35 ± 0.95
A60	60-PEPC	117.40 ± 0.55	0.16 ± 0.01	−2.98 ± 0.86
B0	POPC/CHOL 7:3	170.70 ± 2.19	0.31 ± 0.01	−7.52 ± 1.23
B30	30-PEPC/CHOL 7:3	167.80 ± 2.51	0.32 ± 0.01	−7.22 ± 0.56
B60	60-PEPC/CHOL 7:3	156.60 ± 1.03	0.17 ± 0.01	−3.45 ± 0.97
C0	POPC/DPPC/CHOL/POPE/MTMAB 25:25:20:15:15	138.2 ± 0.95	0.09 ± 0.03	+20.5 ± 1.45
C30	30-PEPC/DPPC/CHOL/POPE/MTMAB 25:25:20:15:15	134.7 ± 1.85	0.31 ± 0.12	+20.9 ± 0.98
C60	60-PEPC/DPPC/CHOL/POPE/MTMAB 25:25:20:15:15	134.5 ± 0.28	0.13 ± 0.19	+19.2 ± 1.11

**Table 2:** Average size diameter, height and height/diameter ratio of POPC and 60-PEPC liposomes analyzed by AFM technique.

	POPC	60-PEPC
Diameter (nm)	145.00 ± 37.98	122.78 ± 13.15
Height (nm)	6.04 ± 1.22	9.28 ± 1.80
Height/diameter ratio	0.04	0.08

**Table 3:** Description of the methods used to optimize the encapsulation of rhodamine B using different liposomal formulations.

Method	Procedure
A	Vortexing (10 min, 22 °C)
B	Vortexing (10 min, 40 °C) + 2 sonication cycles (5 min each)
C	Vortexing (10 min, 22 °C) + 2 freeze-annealing-thaw cycles
D	Vortexing (10 min, 22 °C) + 5 freeze-annealing-thaw cycles

## Abbreviations

AFM	atomic force microscopy
CHOL	cholesterol
D/L	dye to lipid molar ratio
DLS	dynamic light scattering
DPPC	1,2-dipalmitoylphosphatidylcholine
EE	encapsulation efficiency
FAME	fatty acid methyl ester
GC	gas chromatography
LUVET	large unilamellar vesicle by extrusion technique
MLV	multilamellar vesicle
MTMAB	myristyl trimethyl ammonium bromide
PBS	phosphate buffered saline
PDI	polydispersity index
PEPC	1-palmitoyl-2-elaidoylphosphatidylcholine
POPC	1-palmitoyl-2-oleoylphosphatidylcholine
POPE	1-palmitoyl-2-oleoylphosphatidylethanolamine
TFA	*trans* fatty acid
TLC	thin layer chromatography
